# AI-enabled obstetric point-of-care ultrasound as an emerging technology in low- and middle-income countries: provider and health system perspectives

**DOI:** 10.1186/s12884-025-07796-6

**Published:** 2025-07-04

**Authors:** Sara Della Ripa, Nicole Santos, Dilys Walker

**Affiliations:** 1 Institute for Global Health Sciences, University of California San Francisco, San Francisco, CA USA; 2 Department of Obstetrics, Gynecology and Reproductive Sciences, University of California San Francisco, San Francisco, CA USA

**Keywords:** Artificial intelligence, Pregnancy, Ultrasound, POCUS, Africa, Antenatal care, Obstetrics

## Abstract

**Background:**

In many low- and middle-income countries (LMICs), widespread access to obstetric ultrasound is challenged by lack of trained providers, workload, and inadequate resources required for sustainability. Artificial intelligence (AI) is a powerful tool for automating image acquisition and interpretation and may help overcome these barriers. This study explored stakeholders' opinions about how AI-enabled point-of-care ultrasound (POCUS) might change current antenatal care (ANC) services in LMICs and identified key considerations for introduction.

**Methods:**

We purposely sampled midwives, doctors, researchers, and implementors for this mixed methods study, with a focus on those who live or work in African LMICs. Individuals completed an anonymous web-based survey, then participated in an interview or focus group. Among the 41 participants, we captured demographics, experience with and perceptions of standard POCUS, and reactions to an AI-enabled POCUS prototype description. Qualitative data were analyzed by thematic content analysis and quantitative Likert and rank-order data were aggregated as frequencies; the latter was presented alongside illustrative quotes to highlight overall versus nuanced perceptions.

**Results:**

The following themes emerged: (1) priority AI capabilities; (2) potential impact on ANC quality, services and clinical outcomes; (3) health system integration considerations; and (4) research priorities. First, AI-enabled POCUS elicited concerns around algorithmic accuracy and compromised clinical acumen due to over-reliance on AI, but an interest in gestational age automation. Second, there was overall agreement that both standard and AI-enabled POCUS could improve ANC attendance (75%, 65%, respectively), provider–client trust (82%, 60%), and providers’ confidence in clinical decision-making (85%, 70%). AI consistently elicited more uncertainty among respondents. Third, health system considerations emerged including task sharing with midwives, ultrasound training delivery and curricular content, and policy-related issues such as data security and liability risks. For both standard and AI-enabled POCUS, clinical decision support and referral strengthening were deemed necessary to improve outcomes. Lastly, ranked priority research areas included algorithm accuracy across diverse populations and impact on ANC performance indicators; mortality indicators were less prioritized.

**Conclusion:**

Optimism that AI-enabled POCUS can increase access in settings with limited personnel and resources is coupled with expressions of caution and potential risks that warrant careful consideration and exploration.

**Supplementary Information:**

The online version contains supplementary material available at 10.1186/s12884-025-07796-6.

## Background

In 2016, the World Health Organization (WHO) released comprehensive guidelines for routine antenatal care (ANC) [1]. These guidelines include a recommendation for one ultrasound scan before 24 weeks to estimate gestational age and improve detection of fetal anomalies and multiple pregnancies. Across several low- and middle-income countries (LMICs), ultrasound has been shown to improve a woman’s experience of care and increase ANC attendance, largely by providing clients’ peace of mind regarding their pregnancy and fetal well-being [2].

Despite this global recommendation, access to obstetric ultrasound is limited in LMICs, particularly in contexts where physicians, specialist care or sonographers are unavailable [3]. One approach to overcoming the shortage of specialists is task shifting or task sharing with nurses and midwives, leveraging point-of-care ultrasound (POCUS) as a portable tool used at the bedside [4, 5]. A systematic review on training of midwives in Africa to perform obstetric ultrasound scan showed that despite heterogeneous modes of training delivery, curriculum content and competency assessments, midwives across various African countries were able to utilize ultrasound successfully [6]. This included, for example, accurate diagnosis of conditions such as non-cephalic presentation and multiple gestation [7], increased confidence in clinical decision-making [8], and changes to referral decisions [9]. However, issues around increased workload and service disruption due to staff turnover/rotations indicated that introduction of ultrasound among midwives would require additional personnel investments [10, 11]. Other challenges, such as ultrasound costs, ongoing support to ensure quality imaging and interpretation, equipment maintenance, and access to consistent resources (e.g., gel, towels, and electricity) also pose significant barriers to widespread implementation [2, 12, 13].

Artificial intelligence (AI)-enabled obstetric POCUS may transform ultrasound access in LMICs. By providing automated image interpretation that can detect obstetric conditions and abnormalities to inform clinical decision-making, AI-enabled POCUS could reduce scanning times, training and quality assurance approaches, and potentially improve accuracy of assessments [14]. Although AI-enabled obstetric POCUS is not yet available commercially [13] and the ability of an algorithm to perform with high sensitivity and specificity in diverse populations is not yet known, algorithms for some assessments may be ready sooner than others [15]. Several groups have utilized standard plane ultrasonography images and machine learning to estimate gestational age [16–19], while others have explored the detection of other fetal characteristics, such as presentation [17], cardiac structures [20], and neuro-sonography [21]. Recently, promising studies have shown that an AI model to estimate gestational age obtained by a series of blind sweeps across the abdomen is as accurate as trained sonographers’ estimate using fetal biometry, as well as when compared to a cohort with known dating via in vitro fertilization [18, 22, 23].

Once AI algorithms are validated for accuracy, AI-enabled ultrasound devices may have many advantages compared to standard POCUS [24]. For example, this new technology may be superior for identifying high-risk obstetric conditions that may be missed by inadequately trained personnel or in settings where the provider to client ratio negatively impacts quality of care. It may also enable use in remote settings, potentially improving early identification of complications for women in hard-to-reach areas or facilitating gestational age surveillance at the community level. However, many concerns with AI exist, such as applicability of algorithms across diverse geographies and the complexities associated with AI’s ability to learn and adapt models (e.g., the black box) [25, 26]. Logistically, compatibility with existing low-cost POCUS devices will rely on manufacturer characteristics, such as program interfaces and ability to update software applications [13]. A recent survey of LMIC providers suggested perceived utility of an AI-assisted device, particularly around gestational age estimation and fetal viability, though concerns around cost, misdiagnosis and unfamiliarity with the technology were raised [27].

Therefore, while this innovation has game-changing potential to improve global access to ultrasound in LMICs, the introduction of AI-enabled POCUS has varied implications at the individual, provider, and health system level. Questions remain about the technology and its effectiveness, acceptability, and unintended consequences. The objective of this study was to explore stakeholders’ opinions about how AI might transform the current obstetric ultrasound ecosystem in LMICs and to identify key considerations for introduction in these contexts. Given that POCUS task sharing efforts have targeted nurses and midwives [6], this study includes a focus on this cadre of frontline workers.

## Methods

### Study design

We employed a mixed methods approach that included a self-administered quantitative survey, qualitative in-depth interviews and focus group discussions (IDIs and FGDs, respectively). We explored: (1) perceptions about current benefits and challenges with standard POCUS implementation; and (2) opinions about how this ecosystem might change with the introduction of a proposed AI-enabled POCUS prototype.

### Participants

The sample included individuals who work or live in LMIC contexts and have been involved in maternal health, with at least some knowledge of obstetric ultrasound. We focused specifically on individuals who have worked in Africa, in part due to language restrictions (English and French) and that POCUS implementation in African contexts has been well-documented [6]. Participants included nurses, midwives, OBGYNs, doctors, medical officers, researchers, implementors, and funders. We invited individuals who have lived or worked in Kenya, Uganda, Nigeria, Burkina Faso and Zambia. We excluded industry developers to assure device agnostic input. We used purposeful sampling to assure diversity across this sample, based on existing professional networks and authors identified during a parallel scoping review effort.

### Data collection

Standard template emails were sent to invite individuals to participate in the study. Respondents who agreed to participate were asked to fill out a brief, anonymous REDCap survey (Supplemental File) prior to joining either an IDI or FGD. The REDCap survey was intended to quantitatively explore the domains described in Table 1 using both Likert scale and rank-order questions. Electronic consent was obtained at the start of the survey. If applicable, email correspondences was translated into French using ChatGPT (version 3.5) while respondents used Google Translate for the survey based on their web preferences.

**Table 1:** Stakeholder survey and interview domains

Domain #	Area of inquiry	Illustrative questions
Domain #1	Demographics	• Profession • Years of experience • Region(s) of relevant work and residence
Domain #2	Perceptions of standard POCUS	• Familiarity with POCUS • Opinions about POCUS impacts on ANC quality, clinical outcomes, operations, health systems
Domain #3	Perceptions of AI-enabled POCUS* *standard prototype introduced during interviews and focus groups	• Priority AI capabilities • Potential impact on ANC quality and services, clinical outcomes • Considerations for implementation and health system integration • Unintended consequences • Evidence needed and research priorities

For qualitative data collection, we used semi-structured IDI and FGD guides (Supplemental File) to cover the same domains as in the REDCap survey (Table 1). Because the objective of this study was to explore perceptions about AI-enabled POCUS specifically, approximately three-quarters of the time was spent on Domain #3 following initial introductions and questions about prior experience with and perceptions of standard POCUS. Before the AI-focused questions, a standard script describing an AI-enabled POCUS prototype was read by the facilitator:

“*The AI-enabled ultrasound probe is a portable handheld device with screen (smartphone or tablet) that will enable a provider to conduct blind sweeps (three vertical and three horizontal sweeps) across the belly. These sweeps are guided by real-time correction by the device (when the sweep is sufficient, the provider is guided to the next step. If the sweep is not sufficient, the provider is guided to redo the sweep correctly). The image is automatically made available and visible on the screen, along with key information that is automatically generated by AI algorithms. This information will include gestational age dating, singleton or multiples, placenta position, amniotic fluid, and fetal lie.”*

This prototype of a future product was introduced to ensure a shared understanding of base features and was followed by specific questions to delve deeper into participants' impressions. Timing or location of use (e.g., in facilities versus community, during routine ANC versus maternity ward) were not part of this standard description, leaving respondents to envision and prioritize use cases in their individual settings.

Clinical providers were invited to participate in FGDs to promote discussion about usability, where agreement or disagreement could be elaborated on by the group. Providers were grouped by cadre – physicians or midwives—to minimize the impact of professional hierarchies allowing participants to feel more comfortable in expressing their opinions. Decision-makers and implementors were asked to participate in IDIs to explore their motivations and potential barriers to adoption in greater depth. Researchers were invited to either IDIs or FGDs based on scheduling availability, resulting in one mixed clinician-scientist FGD being held (i.e., a nurse, medical officer, and OBGYN who conduct research). In total, six FGDs with 2–7 participants each and 14 IDIs were conducted; three FGDs comprised only African midwives.

IDIs and FGDs were conducted via Zoom and lasted 45 min and 60 min, respectively. Sessions were video-recorded after receiving verbal consent from participants. One member of the research team facilitated the discussion using a semi-structured guide, while one team member served as the primary note-taker. All discussions were conducted in English, except for one midwife FGD in French (translator present). IDIs and FGDs were conducted until data saturation was reached.

### Data management and analysis extraction

To minimize the risk of loss of confidentiality, all video recordings and data were kept on a password-encrypted cloud-based server (i.e., UCSF Box) with restricted access to only study staff.

Survey data were exported from REDCap to Excel and summarized by descriptive statistics. Survey responses were anonymous and presented as aggregate results. All IDI and FGD notes were coded by one of the researchers (SDR). A codebook was iteratively developed for thematic analysis with input from the research team. While initial qualitative notes were associated with identifiable information, this was redacted from transcripts and audio file names. Participant study number and descriptors were used, which included profession, FGD or IDI, and context (LMIC or high-income country, HIC). Direct quotes were extracted from transcripts generated by Zoom and by Descript [92.0.0]. The survey and IDI/FGDs aimed to capture data from the same sample; however, because the former was anonymous, linking information across data streams was not possible.

### Ethics approval and consent to participate

The study was approved by the University of California San Francisco Institutional Review Board (#24–40800). All respondents provided electronic informed consent prior to completing the survey and verbal informed consent prior to participating in IDIs or FGDs.

## Results

### Demographics

In total, 70 individuals were invited to participate. The response rate among midwives was 52.9% (18/34) and 63.9% among other individuals (23/36). Forty individuals completed the REDCap survey, while 41 participated in IDIs or FGDs, indicating that one person did not complete the survey.

Among the survey respondents, most were healthcare providers, with 42.5% midwives/nurses and 20% physicians (Table 2). Over 70% of participants had more than 10 years of experience in the obstetrics field. While approximately two-thirds were LMIC residents—specifically from Kenya, Uganda, Nigeria, Burkina Faso and Zambia—those that did not currently live in an LMIC have worked extensively in these contexts. Three-quarters were familiar with POCUS (i.e., had first-hand experience or were conceptually familiar), and 55% had prior knowledge of efforts in the AI-enabled ultrasound space before participating in this study.

**Table 2:** Demographics of survey respondents (*n* = 40)

	n	%
**Primary profession**		
Physician^a^	8	20.0
Midwife or nurse	17	42.5
Academia/researcher	8	20.0
Implementor/policy/government	3	7.5
Philanthropy/private sector	2	5.0
Not stated^b^	2	5.0
**Years of experience**		
0–5 years	4	10.0
6–10 years	5	12.5
> 10 years	29	72.5
Not stated^b^	2	5.0
**Current LMIC resident**^c^		
Yes	26	65.0
No	12	30.0
Not stated^b^	2	5.0
**Prior POCUS experience**		
No familiarity	8	20.0
Familiar, but no hands-on experience	7	17.5
Familiar, clinically and/or research	23	57.5
Not stated^b^	2	5.0
**Prior knowledge of AI-POCUS**		
No familiarity	14	35.0
Familiar, but no hands-on experience	17	42.5
Familiar, research and/or product development	5	12.5
Not stated^b^	4	10.0

^a^OBGYN, emergency medicine, radiology, medical officers

^b^Respondents declined to fill out demographic domain, but completed the rest of the survey

^c^Some individuals who responded no to residing in an LMIC are LMIC-born but currently reside in another country—using qualitative demographics, 78% (n = 32) were LMIC residents or citizens

The following sections present quantitative and qualitative results related to perceptions of standard and AI-enabled POCUS (Domains 2 and 3, respectively – Table 1) to compare and contrast opinions of the existing and emerging technology.

### Priority AI capabilities

Respondents were asked to rate via a Likert scale the importance of select maternal and fetal assessments that would be most helpful for AI-enabled POCUS to automatically screen for in a basic emergency obstetric and neonatal care (BEmONC) facility (Table 3). Fetal heart rate/viability, multiple gestation and placental location were the three most highly ranked conditions—the latter two were included in the described prototype which was introduced after completion of the survey.

**Table 3:** Maternal and fetal conditions ranked by importance for AI-enabled POCUS to automatically screen for (% of respondents by Likert rating)

	**Very important**	**Somewhat important**	**Neutral**	**Somewhat unimportant**	**Not at all important**	**No answer**
Fetal heart rate/viability	87.5	5.0	2.5	0.0	0.0	5.0
^a^Multiple gestation	85.0	12.5	0.0	0.0	0.0	2.5
^a^Placental location	85.0	7.5	2.5	0.0	0.0	5.0
Ectopic pregnancy	82.5	7.5	5.0	2.5	0.0	2.5
^a^Fetal presentation	80.0	15.0	0.0	0.0	0.0	5.0
Fetal well-being	80.0	12.5	5.0	0.0	0.0	2.5
^a^Amniotic fluid volume	80.0	12.5	2.5	2.5	0.0	2.5
Fetal growth	75.0	20.0	0.0	2.5	0.0	2.5
Confirmation of pregnancy	75.0	12.5	0.0	5.0	2.5	5.0
^a^Gestational age	70.0	22.5	2.5	0.0	0.0	5.0
Placental implantation characteristics	60.0	20.0	12.5	5.0	0.0	2.5
Congenital anomalies	57.5	25.0	7.5	7.5	0.0	2.5
Pre-eclampsia risk prediction	55.0	15.0	17.5	5.0	5.0	2.5
Doppler flow in the umbilical artery	40.0	32.5	17.5	5.0	2.5	2.5
Prior C-section scar integrity	32.5	25.0	22.5	12.5	2.5	5.0

^a^included in the prototype described during IDI/FGDs after completion of the survey

Qualitatively, the prototype capabilities presented to respondents was seen as acceptable:

*“That's some of the biggest risk which we identified: lie or the presentation is one of the risks, and number of fetuses is another risk, and the placenta location is another big risk, which we identified among several others.” – Researcher 2, IDI, LMIC*

While only 70% of respondents felt gestational age – another prototype feature and part of the WHO recommendation—was very important, an additional 22.5% of respondents felt it was somewhat important. Gestational age was flagged during conversation as an important component:

*“I think this artificial intelligence will help us more because, first of all, it has the gestational age. Most of our mothers, a big percentage, are not very sure of their dates, so it's going to really help us.” – Midwife 10, FGD, LMIC*

However, getting women into ANC earlier was a key consideration:

*“Our colleagues think this is really critical to have gestational age [...] but we have to get women in earlier in order to get the most accurate dating.” – Funder/Nurse-midwife 1, FGD, HIC*

Several respondents flagged detection of congenital anomalies as both important and concerning, particularly in LMIC contexts. While ethical concerns were raised about screening for conditions that healthcare providers and systems may not be prepared to manage, others argued for the importance of screening for congenital anomalies to allow clients the option of termination if desired.

*“I think that [detecting congenital anomalies with AI] is also a slippery slope [...] I think it would be really hard if providers had access to that information - that's a whole different type of counseling.” – Policy-maker/Physician 1, IDI, HIC*

*“[With POCUS], the mother gets benefited by knowing that she's going to deliver a live baby… At the same time, also knowing any deformity or congenital abnormality that the baby is having, so that she decides before the time for delivery.” – Midwife 2, FGD, LMIC*

This sentiment was countered with several respondents emphasizing that both standard and AI-enabled POCUS, particularly in the hands of midwives, should be introduced as a screening tool rather than a diagnostic tool.

*“My thinking as an obstetrician, [...] if there's any doubt, then that will be an indication to send this patient [to a] radiologist to rule out the abnormality that you are worried about. So, I think AI will be vital for screening, but we shall need a detailed scan just for confirmatory tests to make sure we reduce on the issue of over treatment.” – OBGYN/Researcher 2, FGD, LMIC*

### Potential impact on ANC quality, services and clinical outcomes

#### ANC utilization and experience of care

**Fig. 1:**
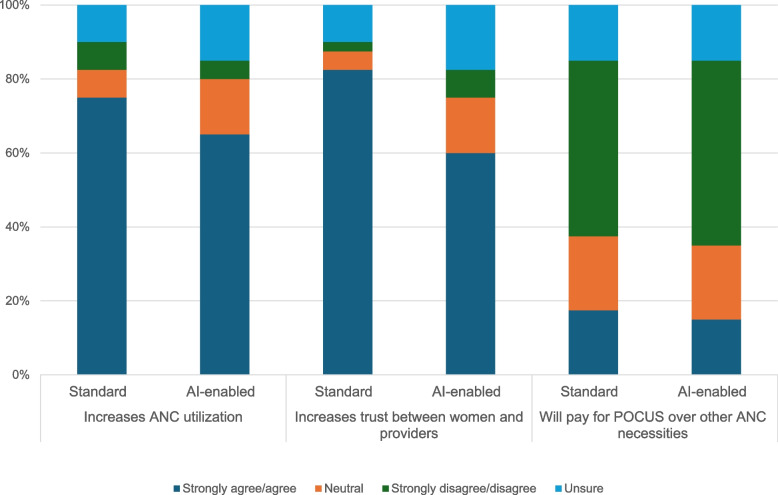
Perceptions of standard and AI-enabled POCUS on ANC utilization and experience (n = 40)

Survey respondents were asked about their perceptions regarding how standard POCUS and AI-enabled POCUS might impact ANC utilization and experience of care. When comparing their survey responses, there was overall strong agreement that both technologies could improve ANC attendance (75% standard and 65% AI), though there was less agreement with AI-enabled POCUS. Fewer respondents felt that AI-enabled POCUS would increase trust between providers and women compared to standard POCUS (60% and 82.5%, respectively). Approximately half of respondents largely agreed that neither technology would lead to clients deciding to forego other ANC necessities in order to pay for a scan. Across these questions, there was slightly more uncertainty with AI-enabled POCUS (Fig. 1).

Findings from IDI/FGDs provided some nuance to these survey data. For example, several respondents believed that AI-enabled POCUS could be seen as less engaging to clients particularly if a clear image of the fetus was not readily available. This could impact ANC utilization, demand and provider–client trust as compared to standard POCUS:

*“Ultimately, a mother wants to see as much as possible of what their baby looks like, and so restricting the images that the nurse gets out of these blind sweeps, I’d say to some extent, [is] a bit limiting to what the mother might want to be seeing and what the nurse might want to be sharing with the mother.” – Implementer 2, IDI, LMIC*

*“And they will be asking us questions like, ‘So that is the only thing that you've done and you're telling me everything is okay? I've not seen you looking for the head.’ [...] But for this new thing, we are not going to be showing them and it's just going to be displaying itself there. I think they'll be asking us a lot of questions…” – Midwife 3, FGD, LMIC*

Respondents noted that the time spent with a client during a standard POCUS scan was critical for human connection and that AI-enabled POCUS may compromise this.

*“For me, I think it'll [AI-enabled POCUS] reduce physical contact with the patient - doctor-patient relationship may be minimal. [...] if it is minimal, sometimes you are not able to explain well, the condition of the patient to the client. And they may feel they are not getting enough information also.” – Midwife 8, FGD, LMIC*

#### Quality and content of ANC services

**Fig. 2:**
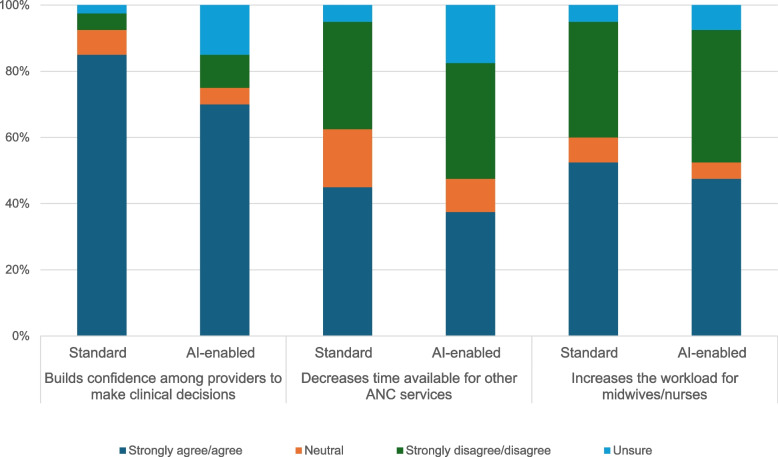
Perceptions of standard and AI-enabled POCUS on client services (n = 40)

The majority (85%) of respondents agreed that standard POCUS can strengthen health care providers’ ability and confidence in making appropriate clinical decisions; however, 70% felt AI-enabled POCUS would build confidence (Fig. 2). Thus, there was more uncertainty with AI-enabled POCUS in reinforcing confidence compared to standard POCUS, a sentiment that was reinforced by qualitative findings.

Some providers tended to express enthusiasm for AI, believing it could enhance accuracy and usability:

*“Those simple sweeps [that are] reproducible and reduce chance of making error is a very good way to go. If those sweeps done correctly can give you, at least those very basic outcomes that you want to know: the presentation, the placental position, and so forth, it'll be useful at least to make that quick decision at a point of care.” – OBGYN/Researcher 3, FGD, LMIC*

*“Yeah, that one will be just a very good help because it is not going to disturb us - like previously, we used to look for the presentation and you move with the probe all over the mother's abdomen looking for one thing, but now this time around we are just going to use it three times and the things will just display. It's just going to be very easy for us.” – Midwife 3, FGD, LMIC*

On the other hand, some respondents expressed concern that clinical acumen may diminish with increased reliance on AI and negatively affect clinical decision-making and confidence.

*“Good midwifery skills are all about hands on bellies, and all about interaction with the woman and all about having a dialogue. [...] I just worry a little bit that if you then put this great machine in the middle of all that, then the emphasis is on the machine. And that if the machine's broken, how will the health worker have maintained their skills and be able to do just hands on belly to work out where the baby is and potentially what size it is. [...] But there are sets of skills that midwives and OBs develop over time which are critically important to maintain.” – Funder, IDI, HIC*

There were more mixed perceptions around whether it would decrease time available for other services and how it would impact provider workload with 30–40% either strongly agreeing or strongly disagreeing with the statement (Fig. 2). A few respondents raised the issue that introduction of POCUS could replace time spent completing another critical ANC components:

*“What are you not gonna do? I feel like in this whole prioritization conversation, nobody says, ‘well, if you're prioritizing something new, you have to deprioritize something’, and we never, ever acknowledge that. So, it means those things get dropped randomly, which means that if the midwife is now prioritizing her time on AI ultrasounds, that she can do, what is she not doing? And if we don't provide guidance on that, she's gonna drop whatever the hardest thing to do is, and that might be the one thing that would save more lives [...].” – Funder/Nurse-midwife 2, FGD, HIC*

While some said poorly remunerated nurses and heavy workloads could de-motivate scanning, others mentioned that AI-enabled ultrasound could mitigate this by decreasing the amount of scanning time needed per client. Several midwife respondents acknowledged the workload, but this sentiment was outweighed by their excitement for the new technology and its potential to allow for more equitable access to ultrasound.

*“Of course the workload will there, but I will [prefer it] this way - I am very okay to do it.” – Midwife 3, FGD, LMIC*

*“You go to the next client, you may conduct the ultrasound to as many clients as possible. Unlike the [standard] POCUS, we sometimes do five to eight [scans]. But for this one [AI-enabled POCUS], you may conduct the ultrasound to almost every client because the results are very fast. I think it's a game changer also to me.” – Midwife 9, FGD, LMIC*

#### Referrals and clinical outcomes

**Fig. 3:**
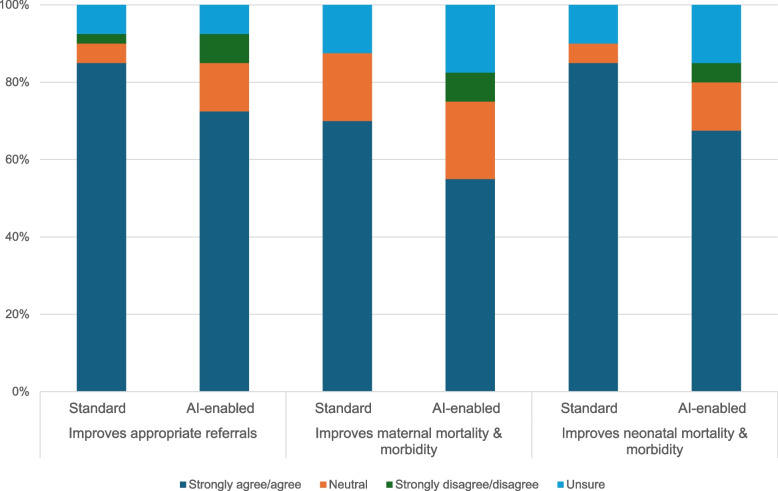
Perceptions of standard and AI-enabled POCUS on referrals and clinical outcomes (n = 40)

The majority of survey respondents felt both technologies could improve appropriate referrals (85% and 72.5% for standard and AI, respectively) and neonatal outcomes (85% standard and 72.5% AI) – and to a lesser degree, maternal outcomes (Fig. 3).

Qualitatively, respondents emphasized that without strengthened and functional referral systems, it is unlikely that either standard or AI-enabled POCUS will have any impact on clinical outcomes.

*“We have to take like a giant step back. [...] There needs to be forward thinking of the referral pathway. [...] Because, what I've seen is that introduction of point of care ultrasound by itself, even in my experience, is never the thing that works by itself. [...] I'm one of the biggest proponents of POCUS, but even more so, I'm one of the biggest proponents of a reminder that POCUS is just a tool in the larger toolbox of clinical tools that we have, including your physical exam and your clinical gestalt of a patient's disease process.” – Researcher/Physician 3, IDI, HIC*

*“When you have added the ultrasound, what do the women do? Where do they go? So I think that we need to really consider how this is embedded within the system and the modifications that need to happen within the clinical pathway for it to be feasibly and sustainably integrated.”* – *Funder/Policy-maker, IDI, LMIC*

*“The purpose of the ultrasound is to identify potential problems and then deal with them appropriately. That’s all part of the intervention. So going back to referrals – I think that has to be front and center. You identify and then you have to know what to do with what you have identified. That’s not a small part of it either.” – Radiologist/Physician, IDI, HIC*

Some respondents expressed concern that referrals would increase overall, potentially straining systems and depleting family resources.

*“If someone gets an ultrasound and they misinterpret something and it leads them to refer a patient that they otherwise wouldn't have referred [...], the process of referring someone is a pretty resource intensive one. When you're coming from a little clinic, and now all of a sudden you have to put someone in an ambulance or you have to tell them you have to mobilize your own resources to go to a hospital that's like, you know, 70 kilometers away to be seen for this thing; they have to go mobilize their own money, a lot of people don't have insurance, so you have to get your own transportation, you have to go pay for the extra test, pay for the extra consultation, worry a lot [about] what could be happening to me, only for them to go and find, ‘Oh no, you're actually okay’. I think that's a potential source of harm.” – Researcher/Physician 2, IDI, LMIC*

In addition to increasing inappropriate referrals, another potential unintended consequence related to health outcomes was inappropriate clinical management. Several respondents voiced concerns about liability risks involved with missed diagnoses or mismanagement due to algorithmic limitations or inaccuracy:

*“If for example, it makes a wrong impression, and I go in and intervene and I’m in error, do I blame the clinician or you blame the machine? [With] the other one [standard POCUS], the person was the one interpreting the picture and saying, ‘I think from my training it is this’, [but] now the [AI] machine has given you that this baby is distressed [then when you go] and deliver, you deliver a preterm baby. That could give you challenges: ‘the machine told me that it was distressed, but it was not actually’.” – OBGYN/Researcher 4, FGD, LMIC*

### Considerations for implementation and health system integration

#### Target user and potential for task sharing

There was general agreement among respondents that midwives should be the primary target-user of AI-enabled POCUS. In the survey (respondents could select up to 3 provider cadres), midwives and nurses were selected most frequently (97.2%), followed by other doctors (55.6%), and OBGYNs (52.8%).

While the central role of midwives was echoed qualitatively, several others also emphasized the need to ensure doctors/OBGYNs were equally trained.

*“I think that's a great idea because, both in private and in public, midwives are the ones who spend a substantial amount of time with the patients. [...] So, strengthening their capacity and empowering them to do point of care ultrasound, I think that would be very welcome to improve outcomes.” – OBGYN/Researcher 3, FGD, LMIC*

*“If for example, the midwife is able to pick it [AI-enabled POCUS], [then], for me to embrace it, to understand what she has referred to me a patient with certain condition, then I need to also get used to it, such that I'm able to be in the same boat with her. [...Otherwise, I’m] subjecting the patient to another scan.” – OBGYN/Researcher 4, FGD, LMIC*

However, some respondents specifically called out AI integration as being more likely to result in professional displacement.

*“So, if everybody and anybody can scan [because of access to AI], then the potential is that people are going to be displaced. And in LMICs, people are looking for jobs. So we don't want professional conflicts arising out of this.” – Funder/Policy-maker, IDI, LMIC*

Relevant to both standard and AI-enabled POCUS, many respondents agreed that provider protections are critical as task sharing of new technologies emerge:

*“I think in terms of training, regulation is not very clear. [...] So I think there is a need for a policy review to ensure that if it is going to be task shifting, then those people are allowed, but also protected of litigation, it's becoming quite common. [...] So I think definitely regulation has to be important; I know sometimes technology comes and these things are rushed because there is a need, but I think looking into that aspect is also important to make sure that there's a policy that is generally agreeable and it define clearly: what one can do and what one can't do. In fact, with the increase in technology, this is becoming quite an important space.” – Researcher/Physician 4, IDI, LMIC*

Introduction outside of the health facility and in the community was met with more heterogenous response. Approximately one-quarter 27.8% of survey respondents felt that community health workers (CHWs) should be trained to use AI-enabled POCUS. Many recognized that CHWs are often the first touchpoint with women, while others voiced concerns related to CHWs’ limited obstetric clinical training and competency to communicate obstetric findings.

*“We are ignoring the fact that women come into contact with the health system in the community. So, we keep trying to incentivize women [...] to come to the facility early. And it really doesn't work. [...] On the other hand, these community health promoters are seeing those women [...]. Let's be very open minded about that first contact, and don't force women to change their behavior. But just leverage what's already there.” – Funder/Nurse-midwife 2, FGD, HIC*

Others believed community-based scanning might de-motivate women from seeking care.

*“The community health promoters are service providers, but they are not well trained in [healthcare]. Their work is to bring us clients as early as possible, like those who are pregnant they should start clinic early. [...] But it [AI-enabled POCUS] is a technical thing which can be used by a provider who can explain more to the client what is happening to the baby.” – Midwife 9, FGD, LMIC*

#### AI-POCUS training

**Fig. 4:**
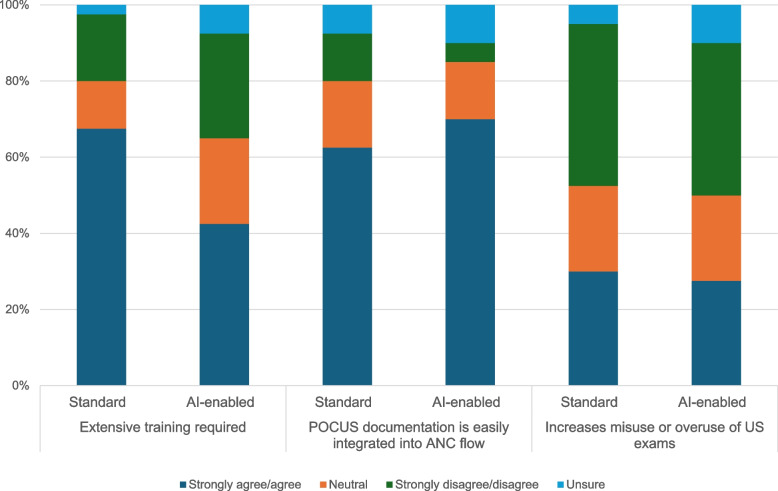
Perceptions of health system integration of standard and AI-enabled POCUS (n = 40)

In terms of training, many believed that AI-enabled POCUS could reduce training requirements compared to standard POCUS training given the ease of blind sweeps. Approximately two-thirds of respondents (67.5%) believed that standard POCUS required intensive training compared to 42.5% for AI-enabled POCUS (Fig. 4).

“*I think that training may not be very complicated, or maybe may not take a lot of time, rather, because the way I see it, it may be easier than [standard] POCUS.” – Midwife 9, FGD, LMIC*

However, several respondents emphasized that concomitant training in POCUS and image interpretation remained critical.

*“But, what knowledge does this type of technology add to me as a provider, because it is just like giving me everything; I'm not even thinking. That may be my worry - a situation where we don't have so much providers who have been trained on ultrasound. Maybe what I would suggest even at the deployment: training on the analog then to the AI, so that at least somebody knows how to interpret images so that even when now I go to this magic gadget that will interpret everything, at least I have something that I've learned from that session.” – OBGYN/Researcher 1, FGD, LMIC*

*“And maybe [with] the AI, it may be easier for them [midwives] to do it maybe in a faster time to be able to get the findings. Except that I believe that training is very important because having the background of POCUS would also be very helpful even as they use the AI [...] I think it's also good to understand, how will the midwife confirm that this machine has told me the right thing?” – Nurse-educator/Researcher, FGD, LMIC*

At least one participant saw AI-enabled POCUS as an opportunity to enhancing existing training programs and focus on strengthening clinical-decision making:

*“Because of the fact that the training itself of the use of ultrasound might be a little bit more facilitated by AI, might leave a little bit more time to focus on the ‘what do you do next’ part, which often, to be very honest, isn't always focused on in point of care obstetric ultrasound classes because people are very, very focused on learning the ultrasound and not learning the clinical algorithm, which is ironic because you need to know the second part of that when you're learning the first part.” – Researcher/Physician 3, IDI, HIC*

#### Resources, data systems and facility infrastructure

Similar to standard POCUS, issues around device maintenance, availability of consumables, machine cost, misuse and overuse (e.g., fetal sex determination, over-charging clients for unnecessary scans, undue anxiety among clients) were mentioned in IDIs/FGDs. In the survey data, there was little to no difference between how either technology might increase ultrasound misuse or overuse with approximately 40% strongly disagreeing with this statement (Fig. 4); however, respondents offered some ideas for how AI might change this, such as disabling identification of fetal sex through “digital diapers.”

*“In terms of the guarding against the misuse for fetal sex detection, and all the consequences of that, where such a limited visualization is desirable, but, on the other hand, the blind sweep paradigm is very restricted in terms of what it can detect.” – Implementer 2, IDI, LMIC*

In the survey data, there was a slight difference in perceptions of how the technology might affect health system documentation with 70% of respondents believing that AI-enabled POCUS would streamline documentation compared to 62.5% for standard POCUS) (Fig. 4). However, many respondents raised larger issues around data privacy and storage, including conflicting interests between industry and governments:

*“[Data storage] is going to become really, really important in the implementation because countries will be like, ‘I don't want my data to go to these U.S. companies’ cloud’. And the more the more you know about it, even with regulations, what you think is anonymous, it can be so easily de-anonymous.” – Policy-maker/Physician 2, IDI, HIC*

*“How well can we localize some of this data [...while still feeding] into the larger technology companies? Because obviously, I know my Minister of Health won't have the capacity to develop some of these technologies - it needs either big corporations or big funders. But now it goes to the ethics part of AI in terms of how well the data is kept, how well all these policies -all these laws- that have been put in place, are being complied to.” – Implementer 1, IDI, LMIC*

In terms of overall health system integration, many respondents emphasized that ultrasound was just one part of the continuum of care, underscoring the need to strengthen systems more broadly.

*“…it can't be called a game changer, per se, without other aspects like, strengthening the system, improving referral, and improving skills, improving availability of commodities, improving emergency obstetric care, but surprisingly also improving the welfare of women.” – Researcher/Physician 4, IDI, LMIC*

*“A lot of the work we've done [...], is to better understand why some of the simpler interventions that are in our guidelines are still not being implemented. For example, why don't we have a blood pressure cuff at every antenatal care clinic that functions? Why don't we have a weighing scale? Why don't we have calcium supplements? So, I think I'm a little bit concerned that if we jumped right to these technological solutions [AI-enabled POCUS], which I know have the potential for huge change, we also might lose on some of the really important interventions that we know are important. It might not be as attractive for providers or for women to pay for.” – Policy-maker/Physician 1, IDI, HIC*

### Evidence needed and research priorities

**Fig. 5:**
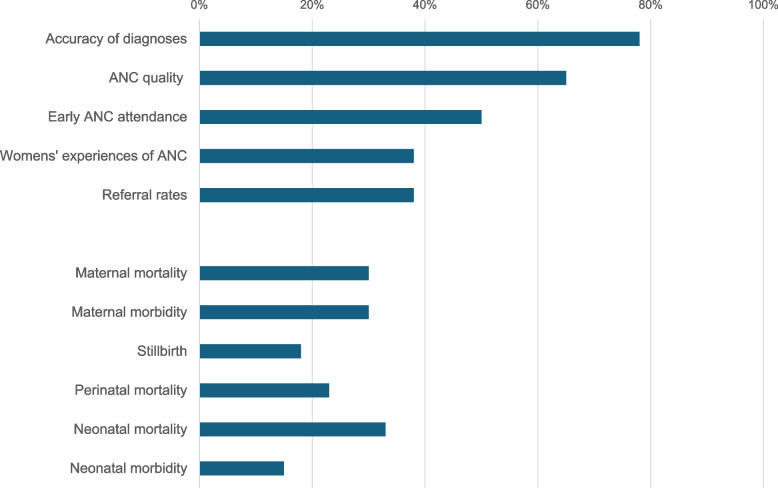
Priority research topics and outcomes related to AI-enabled POCUS (n = 40)

Respondents were asked to prioritize up to five research outcomes for AI-enabled POCUS (Fig. 5) from a list of 12 options. The top five outcomes included accuracy of diagnoses (77.5%), ANC quality (65%), early ANC attendance (50%), impact on referral (37.5%), and women’s experience of care (37.5%).

The importance of accuracy was conveyed by several respondents who cited potential inaccuracies stemming from a lack of diversity in training datasets. To mitigate bias, they emphasized the necessity of using representative and diverse datasets for algorithm training.

*"My only question would be in terms of training these algorithms [...] whether they'll be consistent across the different populations […] so that at least whenever you get an outcome, it is an outcome that can actually help you make a decision, not an outcome that gets you into an error."– OBGYN/Researcher 3, FGD, LMIC*

*“In India it's generally known that a baby is small for gestational age, but may not be necessarily a preterm, there are more small babies. And then in Africa, there are bigger babies. So this algorithm has been tested on who? Does it work? Where does it work?” – Researcher/Physician 4, IDI, LMIC*

Priorities related to ANC reflect the need to assess impact on ANC quality, early ANC attendance, and women’s experience of care during the pregnancy journey.

*“I would want to, to answer the question: does it improve referrals that we're getting over the analog? [...] So on the part of the provider, do they think it's better because it's taking less time? [...] We have challenges with human resources in our facilities. [...] Does it improve the time they are taking to provide the service, but at the same time, not compromising quality of the work?” – Researcher 3, IDI, LMIC*

*“And how do we really assess what is good counseling and what does that that mean? So, I mean, if there's an opportunity with all this excitement about AI ultrasound to really do some research around how do we ensure effective counseling around communicating what it means, and then how does the woman then interpret that and use that also for her own discussions with her, her family. [...] I think there's a lot of great questions that could be, that could be looked at more on some of the operational issues.” – Policy-maker/Physician 1, IDI, HIC*

Measures related to maternal mortality and morbidity, stillbirth, neonatal mortality and morbidity were less prioritized (selected by 15–32% of the survey sample) than more proximal outcomes.

*“I think we don't need studies that look at, ‘Does it reduce maternal mortality? Does it reduce newborn mortality? [...] We should look at earlier outcomes: ‘Is it diagnosing things correctly? Is it leading to correct referrals?’ I think that's the kind of studies we should be designing. And of course, ‘What does it mean to implement it within a health system?’” – Policy-maker/Physician 2, IDI, HIC*

*“My point would be to think through what are these main causes of adverse maternal outcomes and birth outcomes. In our setting, we know it's PPH, preeclampsia, sepsis, and so forth. Then see how we can integrate the tool towards mitigation of those big five. So, if we have that integrated within the tools, then we might see some level of reduction in the adverse outcomes. If not, then we'll just still have the status quo.” – OBGYN/Researcher 3, FGD, LMIC*

## Discussion

Through robust engagement with a diverse group of stakeholders, this work provides valuable insights to inform the potential future of AI-enabled POCUS in LMICs. Overall, there was broad excitement and optimism about the potential of AI to transform POCUS access for pregnant women in settings with limited personnel and resources. However, positive anticipation was tempered by expressions of caution, unanswered questions, and potential disruptions that warrant further exploration and careful consideration. As just one potential ANC tool, AI-enabled POCUS was not seen as a silver bullet – instead, its implementation must consider the health system in which it is deployed to assure overall improved quality of ANC across the continuum of care.

**Fig. 6:**
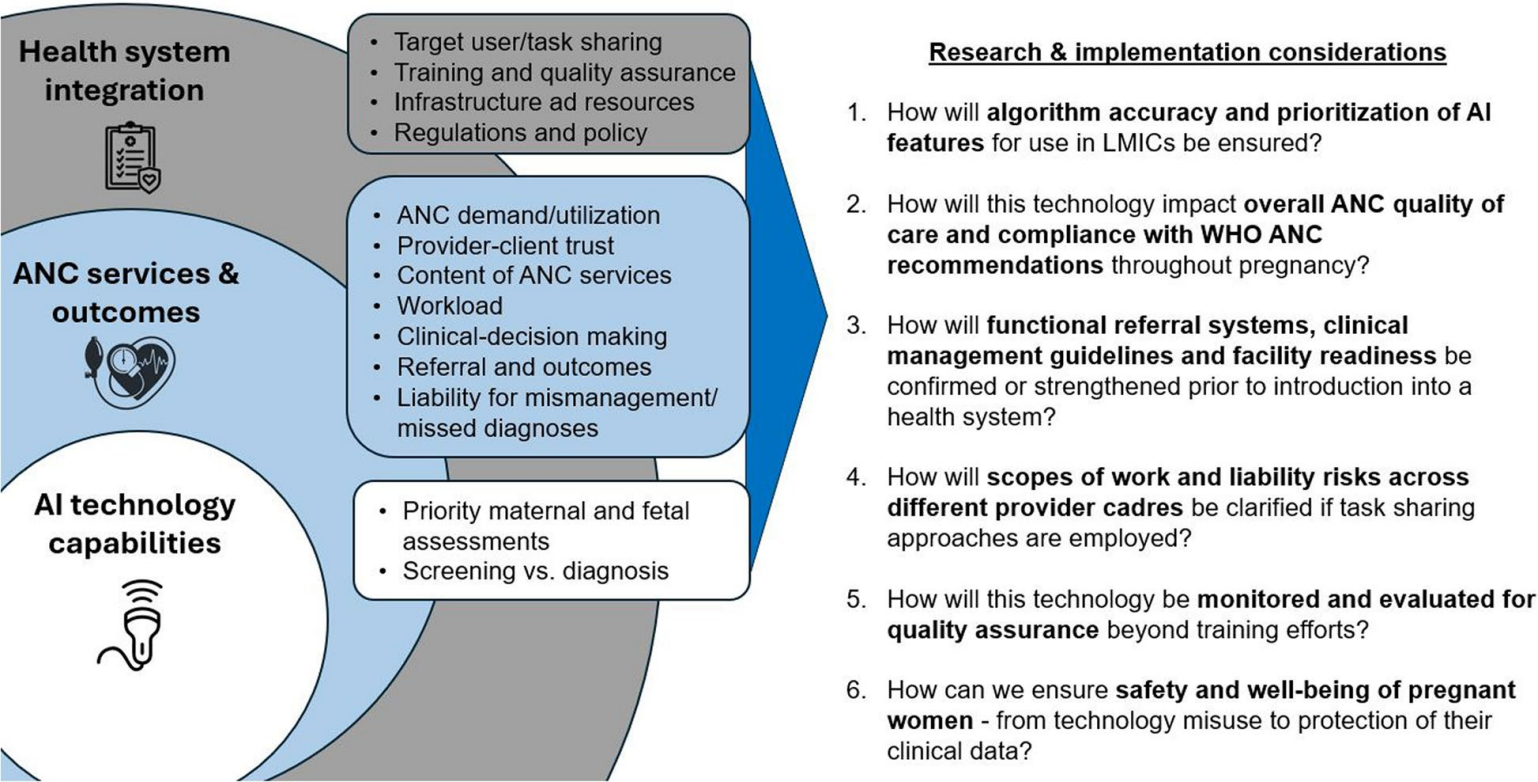
Summary of key considerations for introduction of AI-enabled obstetric POCUS in LMICs

Together, our qualitative and quantitative findings mapped to three key areas: 1) input on the AI technology capabilities, 2) potential impact on ANC services and outcomes, and 3) integration with the overall health system. While this emerging technology has the ability to greatly shift this ecosystem, we also acknowledge that much research remains to be conducted to ensure responsible and equitable introduction and implementation. Additionally, the need for clear policies and regulations – for example, those related to data privacy and management, tasking shifting and clinical liability, screening and referral, and training certification – emerged as an over-arching theme. Figure 6 provides a synthesis of these findings and highlights some priority research and implementation considerations that emerged.

*How will algorithm accuracy and prioritization of AI features for use in LMICs be ensured? *In terms of AI capabilities, there was much enthusiasm for maternal and fetal assessments that could be automated, respondents emphasized the need to validate algorithm sensitivity and specificity prior to introduction and contextualize use in LMICs. For example, accurate gestational age dating by AI could be influenced by late ANC seeking behaviors and geographically varied fetal growth patterns [28, 29]. Ethical considerations were also raised for assessments that are generally outside the current clinical or counseling scope of LMIC midwives (e.g., congenital anomalies), as well as how inappropriate referrals could cause financial strain on some families. The latter sentiment has been raised in standard POCUS-related studies [30, 31] and perhaps amplified with the prospect of machine-driven interpretation. These concerns underscore the importance of clearly differentiating device use for screening for referral to specialty care or a second opinion versus diagnosis.

*How will this technology impact overall ANC quality of care and compliance with WHO ANC recommendations throughout pregnancy? *One critical question raised was whether overall ANC quality will remain the same, improve, or decrease with the introduction of AI-enabled devices. Many respondents generally felt that both standard and AI-enhanced POCUS would improve ANC utilization, client satisfaction and provider confidence in clinical decision-making; notably, these sentiments have previously been observed in a number of studies exploring POCUS, such as in Ethiopia, Kenya and Uganda [10, 32–37]. However, concerns around reduced client interaction and engagement and over-reliance on AI outputs were mentioned.

While there are various studies that have looked at how POCUS affects ANC delivery [35, 38, 39], robust quantitative evidence regarding what happens to overall ANC quality when you introduce POCUS to an already stretched system is lacking [40]. Given the limited time available for ANC visits in LMIC contexts, some respondents acknowledged that other important services may be overlooked or de-prioritized, while others – including several midwives – felt this would not exacerbate workload or impact provision of other services. Additionally, introduction of POCUS or AI-enabled POCUS will require health system expenditure. In settings where many providers are struggling with access to critical supplies and commodities, such as blood pressure cuffs and iron/folic acid supplements, there is risk that provision of comprehensive ANC will suffer. Thus, the risk of exacerbating inadequate content of care or poor compliance with existing ANC recommendations must be addressed, ideally using standardized monitoring indicators so that lessons can be learned across contexts [41].

*How will functional referral systems, clinical management guidelines and facility readiness be confirmed or strengthened prior to introduction into a health system?* In many LMICs, the current status of clinical decision-making, referral systems, and documentation is inadequate [42–44] – hindering the potential for standard or AI-enabled POCUS to have clinical impact. Respondents highlighted how health system issues must be addressed concurrently, such that all ANC services and systems are strengthened. This idea, along with consistent provision of supplies, device maintenance and cost, are consistent with implementation considerations related to standard POCUS, as well [2, 13, 45]. Conceptually, the transition to AI automation and interpretation could increase scanning volume, and thus the resources required by the facility, underscoring the importance of addressing sustainability early in the introduction phase.

*How will scopes of work and liability risks across different provider cadres be clarified if task sharing approaches are employed? *One surprising finding was the potential for AI-enabled POCUS to negatively impact interprofessional collaboration. While advantages and disadvantages related to both task sharing and task shifting have been explored [5, 6], bringing a machine capable of interpretation into the inter-cadre dynamic is a novel consideration. In our study, some physicians emphasized the importance of including them together with midwives in the initial introduction plan such that they are familiar with the technology to build trust in it and understand its role in decision-making. Though not explicitly stated by respondents, AI-generated findings may be seen as more acceptable by higher-level providers than standard POCUS performed and interpreted by lower-level providers. Therefore, intentional integration of cadres involved in the pregnancy and referral pathways will likely need to be considered in training strategies.

Notably, providers brought to light liability concerns related to the AI device, particularly for midwives. In most health systems, provision of ultrasound services is not defined in midwifery scopes of practice and thus, current policies and absence of training certification do not protect them. Several respondents emphasized that POCUS is a powerful tool that can increase midwife competencies, professional standing, and strengthen their relationships with clients and their families. Depending on the envisioned training and device capabilities, AI-enabled POCUS risks diminishing the professional and personal gains achieved by POCUS. These concerns highlight the potential benefits of a hybrid model that includes an “AI-assist” feature, in addition to the limited functionality of the blind sweep model. While the former may be more suited for facility-based ANC, the latter may be more suited to primary or community level deployment.

*How will this technology be monitored and evaluated for quality assurance beyond training efforts?* According to several respondents, AI-enabled POCUS has the potential to shorten training windows, make it more accessible to more individuals without disturbing workflows, and mitigate issues related to staff turnover and rotations. However, they felt modules related to the fundamentals of ultrasonography are still desired such that trainees feel equipped to interpret images. Additionally, they felt the time saved in training should be used to strengthen clinical decision-making support, referral processes and documentation. Questions arose about what happens after initial training in terms of assuring that users are adequately trained not just in blind sweeps, but also subsequent decisions and actions. This includes monitoring indicators around misuse and overuse, such as those related to fetal sex determination, financially motivated scans, etc.

*How can we ensure safety and well-being of pregnant women—from technology misuse to protection of their clinical data? *We were not surprised to hear about concerns related to the technology itself, including questions about algorithm accuracy, data privacy, and ownership. Engagement with broad populations to optimize data diversity to avoid algorithmic bias must be pursued and in the context of ethical research and implementation. Notably, in our study, the idea of locally or regionally derived algorithms generated by in-country AI developers or industry was not raised, but is worthwhile to consider. While country-specific policy and data regulation undoubtedly play a role, individual stakeholders must also commit to social justice and accountability. These sentiments echo areas of concern raised by many groups including governmental and implementation bodies, as the rapid acceleration of efforts in the broader AI space continue with unclear oversight and guardrails [46, 47].

Given the growing field of AI, respondents were clear that more research is needed. Robust data on accuracy of AI-enabled POCUS across diverse populations was voiced as a priority, as well as intermediate outcomes related to ANC and referral. However, the majority felt focusing on research to assess AI-enabled POCUS at ANC and its impact on maternal and neonatal mortality and morbidity is not a useful allocation of scarce resources for research. Similar to the concerns raised in the First Look Study, sub-optimal health systems and referral pathways would preclude meaningful and generalizable findings [31, 39, 48]. However, we note that the potential use of AI-enabled POCUS during labor triage is an opportunity to assess direct impact of POCUS on maternal and neonatal outcomes, as well as key contributors to mortality, such as unnecessary Cesarean sections and inductions. Abnormal findings discovered at labor triage could lead to modifications in immediate care pathways that impact both maternal and neonatal outcomes. Conversely, exploring the community-based use of AI-enabled POCUS in early pregnancy could achieve more accurate gestational age dating and identify early pregnancy loss which may have benefits.

### Strengths and limitations

Our study has a number of strengths and limitations. The strengths include a diverse group of relevant stakeholder inputs with a majority being clinicians (primarily midwives) with POCUS experience from LMICs. Our mixed methods approach allowed for a more nuanced understanding of perspectives.

Our limitations include a small sample size with skewed input from African stakeholders. Clinician perspectives from other regions would have added health system diversity; for example, some Southeast Asia countries where the role of community health workers is more formally defined would add depth. Additionally, we had little representation from radiologists and sonographer technicians, who may have had varying views on task sharing, impact of hierarchy, and cadre prioritization. And finally, all perspectives related to the AI-enabled prototype are hypothetical, as the device is not yet publicly available, though a standard description was shared to ground assumptions.

## Conclusion

AI-enabled POCUS offers a transformative approach to increase global access to ultrasound during pregnancy. However, there remain unanswered questions, unintended consequences and potential risks that warrant careful consideration and strong policy guidance to limit the potential disruption. Product development and subsequent introduction must center clients, providers and the health system in which it is deployed to assure ANC quality is improved, not diminished.

## Supplementary Information

Supplementary Material 1: Survey: AI-Enabled POCUS for Obstetrics in LMICs.

## Data Availability

The datasets used and/or analyzed during the current study are available from the corresponding author on reasonable request.

## Abbreviations

WHO: World Health Organization
ANC: Antenatal care
POCUS: Point-of-care ultrasound
LMIC: Low- and middle-income country
HIC: High-income country
AI: Artificial intelligence
OBGYN: Obstetrician/gynecologist
IDI: In-depth interview
FGD: Focus group discussion
