# Supplementary material for: AI-enabled obstetric point-of-care ultrasound as an emerging technology in low- and middle-income countries: provider and health system perspectives
Source: BMC Pregnancy Childbirth. 2025 Jul 4;25:729. doi: 10.1186/s12884-025-07796-6 (PMC12229288; doi:10.1186/s12884-025-07796-6)
Supplement: Supplementary file 1 — Supplementary Material 1: Survey: AI-Enabled POCUS for Obstetrics in LMICs. [file 12884_2025_7796_MOESM1_ESM.pdf]

Survey: AI-Enabled POCUS for Obstetrics in LMICs

This survey aims to capture your opinions related to the use of artificial intelligence (AI) to enhance obstetric ultrasound in low- and middle-income countries (LMICs). It includes questions about your background, perceptions about both standard point-of-care ultrasound (POCUS) and AI-enabled POCUS devices, and your experiences with obstetric clients seeking scans. It should take no more than 15 minutes to complete. Your responses will help guide and inform the discussion of the interview or focus group we will invite you to participate in. This work is part of a research study being done by Dr. Dilys Walker at the University of California, San Francisco. Being in this study is optional. If you choose to be in the study, you can skip questions that you do not want to answer or stop the survey at any time. The survey is anonymous, and no one will be able to link your answers back to you. Please do not include your name or other information that could be used to identify you in the survey responses. Questions? Please contact study PI Dilys.Walker@ucsf.edu. If you have questions or concerns about your rights as a research participant, you can call the UCSF Institutional Review Board at +1-415-476-1814.

If you would like to participate in this study, choose "I consent" to start the survey.

- ◯ I consent
- ◯ No

(If no, you may close the survey.)

Section A: DEMOGRAPHIC INFORMATION

We will first gather information about your background.

A.1 What is your primary profession?

- ◯ Clinician
- ◯ Academia/researcher
- ◯ Private sector/industry
- ◯ Policy/Government
- ◯ Philanthropy/Foundation
- ◯ Implementor
- ◯ Other, specify: ______

(Choose one.)

A.1.1 If you are a clinician, please specify:

- ☐ OB/GYN
- ☐ Radiologist
- ☐ Physician, medical officer or clinical officer
- ☐ Midwife
- ☐ Nurse
- ☐ Sonography or radiography technician
- ☐ Other, specify: ______

A.1.2 If you are a clinician, approximately how many antenatal clients do you see in your setting per month?

- ◯ Less than 20
- ◯ Less than 100
- ◯ More than 100

A.2 How many years of experience do you have in global maternal/neonatal health?

- ◯ 0-5 years
- ◯ 6-10 years
- ◯ 11-20 years
- ◯ 20 years or more

A.3 If you work in LMICs, select all regions in which you work or have worked on maternal/neonatal health.

- ☐ Africa
- ☐ South/ East Asia & Pacific
- ☐ Middle East
- ☐ Latin America & Caribbean
- ☐ Other, specify: ______

(Select all that apply.)

A.4 Do you reside in a LMIC?

- ◯ Yes
- ◯ No
- ◯ Decline to state

A.4.1 If yes, in which country?

____

A.4.2 If you are a clinician in an LMIC, what type of health facility do you primarily work?

- ◯ Primary health center
- ◯ District-level hospital
- ◯ Tertiary referral hospital or academic teaching hospital
- ◯ Private clinic or hospital
- ◯ Not-for-profit private/missionary hospital
- ◯ Other, specify: ______

Section B: PERCEPTIONS ABOUT POCUS

We would first like to explore your general perceptions about point-of-care ultrasound (POCUS) which refers to a portable ultrasound machine used by a nurse or midwife in a clinical setting for real-time diagnostic and procedural guidance (i.e. at the bedside).

B.1 Prior to this survey, were you familiar with point-of-care ultrasound?

- ☐ I have been involved in research related to standard POCUS
- ☐ I use POCUS in my clinical duties/scope of work
- ☐ I am familiar with POCUS but do not have first hand use experience
- ☐ I am not familiar with POCUS and this is the first time I have learned about it
- ☐ Other, specify: ______

(Check all that apply.)

B.2 How much to you agree or disagree with each statement below when considering standard POCUS use in LMIC health facilities that provide basic emergency obstetric and neonatal care (BEmONC) (e.g. emergency care without access to C-section or blood products)?

	Strongly disagree	Disagree	Neutral	Agree	Strongly agree	Unsure
POCUS increases ANC utilization	◯	◯	◯	◯	◯	◯
Documentation of POCUS findings is easily integrated into ANC flow	◯	◯	◯	◯	◯	◯
POCUS decreases time available for other ANC services	◯	◯	◯	◯	◯	◯
POCUS increases misuse or overuse of US exams	◯	◯	◯	◯	◯	◯
Extensive training and certification is required for POCUS	◯	◯	◯	◯	◯	◯
POCUS increases trust between women and providers	◯	◯	◯	◯	◯	◯
POCUS improves appropriate referrals	◯	◯	◯	◯	◯	◯
POCUS improves maternal mortality and morbidity outcomes	◯	◯	◯	◯	◯	◯

POCUS improves neonatal mortality and morbidity outcomes	○	○	○	○	○	○
There are significant regulatory hurdles for POCUS machines and supplies	○	○	○	○	○	○
There are significant procurement challenges for POCUS machines and supplies	○	○	○	○	○	○
POCUS increases the workload for midwives & nurses	○	○	○	○	○	○
POCUS builds confidence among midwives/nurses to make decisions	○	○	○	○	○	○
Women will pay for POCUS over other ANC necessities (e.g. iron and vitamins)	○	○	○	○	○	○
POCUS increases husbands/ partners' engagement with the pregnancy	○	○	○	○	○	○

B.3 Concluding Section B
Is there anything else you would like to share or expand on regarding POCUS use/ implementation in LMICs? ____

Section C: PERCEPTIONS ABOUT AI-ENABLED POCUS

The following questions ask for your opinions about AI-enabled POCUS. As you may know, AI technology is in development to enhance ultrasound imaging for detecting various obstetric conditions. When the probe is swept over the pregnant abdomen in a specific pattern (e.g. blind sweeps), the AI-enabled POCUS automatically analyzes and provides critical information on the screen about the pregnancy. This tool aims to support initial screening and decision-making, including timely referrals for specialized diagnostic assessments or advanced care. Currently, this emerging technology is not yet available for clinical use worldwide.

C.1 Prior to this survey, were you familiar with AI-enabled ultrasound?

- ☐ I have been involved in research or product development related to AI algorithm development or validation
- ☐ I have heard about AI applications for obstetric use from a study, clinical trial, website, and/or professional networks
- ☐ I am not familiar with AI-enabled ultrasound OB applications and this is the first time I have learned about it
- ☐ Other, specify: ______

(Select all that apply.)

C.2 Imagine you were using an AI-enabled POCUS device to automatically screen for specific maternal and fetal conditions in LMIC health facilities that provide basic emergency obstetric and neonatal care (BEmONC) (e.g. emergency care without access to C-section or blood products).

How important are each of the following assessments on impacting maternal and fetal outcomes in BEmONC

facilities?

	Not at all important	Somewhat unimportant	Neutral	Somewhat important	Very important
Fetal heart rate/ viability	○	○	○	○	○
Fetal well-being	○	○	○	○	○
Fetal growth	○	○	○	○	○
Fetal presentation	○	○	○	○	○
Confirmation of pregnancy	○	○	○	○	○
Gestational age	○	○	○	○	○
Multiple gestation	○	○	○	○	○
Congenital anomalies	○	○	○	○	○
Placental location	○	○	○	○	○
Placental implantation characteristics	○	○	○	○	○
Amniotic fluid volume	○	○	○	○	○
Doppler flow in the umbilical artery	○	○	○	○	○
Ectopic pregnancy	○	○	○	○	○
Pre-eclampsia risk prediction	○	○	○	○	○
Prior C-section scar integrity	○	○	○	○	○

C.3 Are there are other assessments that you feel are important for AI-enabled POCUS to automatically screen for? If so, please describe them here: ______________________________

C.4 In your opinion, what type(s) of provider should be the highest priority target user(s) of AI-enabled POCUS devices in LMIC health facilities that provide basic emergency obstetric and neonatal care (BEmONC) (e.g. emergency care without access to C-section or blood products)? Choose up to 3.

- ☐ OBGYN
- ☐ Radiologist
- ☐ Other physician/ clinical officer
- ☐ Midwife/ nurse
- ☐ Community health worker
- ☐ Radiography/ sonography technician
- ☐ Other, specify: ______

(You may choose up to 3.)

C.5 How much do you agree or disagree with the following statements when considering AI-enabled POCUS in LMIC health facilities that have basic emergency services (BEmONC), (e.g. maternity care without access to C-section or blood products)?

	Strongly disagree	Disagree	Neutral	Agree	Strongly agree	Unsure
AI-enabled POCUS will increase husbands/ partners' engagement with the pregnancy	○	○	○	○	○	○
AI-enabled POCUS will increase misuse or overuse of US exams	○	○	○	○	○	○

Significant procurement challenges are expected for POCUS machines and supplies	○	○	○	○	○	○
AI-enabled POCUS will decrease time available for other ANC services	○	○	○	○	○	○
Significant regulatory hurdles for AI-enabled POCUS machines and supplies are likely	○	○	○	○	○	○
Women will pay for AI-enabled POCUS over other ANC necessities (e.g. iron and vitamins)	○	○	○	○	○	○
Documentation of AI-enabled POCUS findings will be easily integrated into ANC flow	○	○	○	○	○	○
AI-enabled POCUS will increase ANC utilization	○	○	○	○	○	○
AI-enabled POCUS will increase the workload for midwives & nurses'	○	○	○	○	○	○
Users will require extensive training and certification for AI-enabled POCUS	○	○	○	○	○	○
AI-enabled POCUS will increase trust between women and providers	○	○	○	○	○	○
AI-enabled POCUS will improve maternal mortality and morbidity outcomes	○	○	○	○	○	○
AI-enabled POCUS will improve neonatal mortality and morbidity outcomes	○	○	○	○	○	○
AI-enabled POCUS will build confidence among midwives/nurses to make decisions	○	○	○	○	○	○
AI-enabled POCUS will improve appropriate referrals	○	○	○	○	○	○

C.6 With regard to generating evidence for AI-enabled POCUS in LMICs, which of the following would you prioritize? Choose up to 3.

- [ ] How the device impacts clinical outcomes in a highly controlled research environment
- [ ] How the device impacts process outcomes in real-world settings (effectiveness, like ANC utilization, referral, clinical decision-making)
- [ ] Patient experience, including acceptability, experience of care, willingness to pay, etc.
- [ ] Provider experience, including ease of use, acceptability, adoptability
- [ ] Factors such as cost, training modalities, skill retention, supply chain, scalability (implementation research)
- [ ] Which health system factors are required for effective implementation
- [ ] Nothing, I think AI-enabled POCUS is not a good use of funds or resources
- [ ] Nothing, it should be introduced widely without delay

(You may choose up to 3.)

C.7 Is there other research not listed above that you think is important regarding AI-enabled POCUS implementation in LMICs?

____

C.8 What healthcare system components need to be assessed and intact prior to introduction of AI-enabled POCUS?

____

C.9 What research outcomes would you prioritize? Choose up to 5.

- [ ] ANC quality (provision of care aligned with local guidelines)
- [ ] Womens' experiences of ANC
- [ ] Early ANC attendance
- [ ] Referral rates
- [ ] Accuracy of diagnoses
- [ ] Maternal morbidity
- [ ] Maternal mortality
- [ ] Stillbirth
- [ ] Neonatal mortality
- [ ] Neonatal morbidity
- [ ] Perinatal mortality
- [ ] Congenital anomalies
- [ ] Other, specify: ______

(You may choose up to 5.)

C.10 Concluding Section C
Is there anything else you would like to share or expand on regarding AI-enabled POCUS use/implementation in LMICs?

____

Section D: PERSPECTIVES REGARDING YOUR EXPERIENCE WITH CLIENTS RECEIVING OB ULTRASOUND EXAMS

This section explores your understanding of clients' perspectives about receiving OB US exams in LMIC(s) either in a clinical, research, or other capacity.

D.1 From the literature that we reviewed, we found that women expressed the following perceptions/attitudes/experiences when receiving US scans.

How much do you agree or disagree with each statement when considering womens' perceptions of POCUS?

	Strongly disagree	Disagree	Neutral	Agree	Strongly agree	No experience/ no opinion
Knowing fetal sex is important to women.	○	○	○	○	○	○
There is peace-of-mind for women in seeing the fetus and hearing the heartbeat during US exams.	○	○	○	○	○	○
Limited communication from providers about US procedures and results leads to dissatisfaction and anxiety in women.	○	○	○	○	○	○
Women are confused when gestational age estimates differ between US and other methods.	○	○	○	○	○	○
Women seek repeated exams even when it exceeds the recommended number.	○	○	○	○	○	○
Women are worried about the safety of US scans.	○	○	○	○	○	○

D.2 From the client perspective, how do you think AI-enabled POCUS might change the patient experience and/or opinions as compared to regular POCUS (non-AI-enabled)? ______________________________

Thank you!

Thank you for completing this survey. We hope to synthesize feedback prior to the focus group discussion so that we can further explore these issues. No names will be linked in any way to responses.

In-Depth Interview (IDI) and Focus Group Discussion (FGD) Guides for Stakeholder Engagement

Provider Guide

Thank you for agreeing to take part in this focus group discussion. We would like to add some ground rules before going further. We recognize you are from diverse backgrounds and work in varied settings. We are eager to hear from all of you and all opinions and ideas and perspectives are of value to us. Can we agree each person will raise their hand to speak? You can also use the chat window for additional comments or clarifying questions, if necessary. We hope to have you share your thoughts and respond to the ideas and perspectives of others in a way that is respectful, honest, and comfortable. We would like to record this meeting, is that okay with you?

Start with overview of the project:
We are here today to get your perceptions about AI-enabled point of care obstetric ultrasound (POCUS) in LMICs. This is an emerging field whereby AI is being used to analyze images captured by blind sweeps of the pregnant woman's abdomen. The output could be identification of conditions that might warrant further assessment, attention or higher level of care, such as fetal position, multiple gestation, placental abnormalities, etc.

Before we start, do you have any questions?
Let's go around and introduce ourselves:

- *First name only*
- *How many years in field*
- *Type of facility they currently work at and approximately how many antenatal clients they see a month (if provider-focused group or interview)*

**note that identifying information (e.g., your name) will not be linked to any data presented.*

**Domain**	**Prompts/questions**
Current practice for ultrasound	Let's start by talking about current practice related to use of ultrasound.<br>• Where and how does obstetric US get performed? (Do you have POCUS or do you send clients to another unit?)<br>○ Probe: referral challenges<br>• ANC vs. maternity unit<br>• Perceptions on how it impacts your workflow, workload, etc.<br>• Perceptions on how it impacts your provider-patient dynamic
Existing knowledge and experience with POCUS	For those of you who have POCUS in your setting<br>• What has been the hardest part of using POCUS in your setting?<br>• Tell us about your training? What worked and what didn't work?<br>• What cadre of provider uses POCUS?<br>• Was certification required?<br>• Any mechanisms for quality control?<br><br>What are some issues related to POCUS adoption?<br>• Staff turnover<br>• Costs, supplies, machine maintenance etc.<br>• Women's demand<br>○ Probe: demand by trimester<br><br>How does using POCUS help you make decisions for clinical management of your patients? // Can you think back to the last time you performed an abnormal scan and tell me about what and you patient did to navigate it?

**Prototype Introduction:** Device Description prior to AI-related questions	Disclaimers:<br>1. This AI-enabled component is not currently available in your country<br>2. We are not here to advocate for the technology, but we are here to understand your opinions about its potential implementation<br>3. This technology is not yet cleared for use in any other country however the BMGF is putting effort into making it accessible. We are sharing our findings with them but do not represent BMGF and encourage you to be honest about your opinions about this technology.<br><br>We will now show you/ tell you about how the technology works (PPT/ video/ etc)<br><br>*The AI-enabled ultrasound probe is a portable handheld device with screen (smartphone or tablet) that will enable a provider to conduct blind sweeps (three vertical and three horizontal sweeps) across the belly. These sweeps are guided by real-time correction by the device (when the sweep is sufficient, the provider is guided to the next step. If the sweep is not sufficient, the provider is guided to redo the sweep correctly). The image is automatically made available and visible on the screen, along with key information that is automatically generated by AI algorithms. This information will include:* ***gestational age dating, singleton or multiples, placenta position, amniotic fluid, and fetal lie.***<br>• If asked about the cost: "Cost is certainly a consideration in the product development process, but it's too early in the product development process to share information on target pricing."
How might AI alter this experience?	Given the prototype that we just described, what are the potential benefits?<br>• How will the AI-component improve POCUS for you? For OB services for your patients?<br><br>You mentioned xyz problems with POCUS, how do you think AI will address or not address your earlier concerns with POCUS?<br>• Clinical: identification of complication, referral, case presentation<br>• Provider: turnover, skills retention, ease of use, behavior change<br>• Women: ANC utilization, fear<br><br>Given the prototype that we just described, what are some unintended consequences that might occur with the introduction of AI-enabled POCUS?<br><br>What do you expect will be barriers to implementing or adopting this into your setting?<br><br>Would you try this prototype now if you had access to it?<br>• In what situations would it be most useful?<br>• What resources would need to be in place already in order to implement?<br>• What would be the ideal training approach/modality?<br><br>Given the prototype that we just described, how do you think your clients would respond to information provided by an AI-enabled US compared to the current mode of POCUS?<br>• How important is the image functionality to you and the women you see?<br><br>What research questions/data would you want to see before you would try it?
Other critical factors to consider	What other additional considerations do you think need to be made before or in parallel with implementing the prototype described?<br>• Supply chain, cost<br>• Medical liability<br>• Polices or guidelines<br><br>The prototype that we described is what is currently under development. If you could choose, would you work on an AI device with different functionalities? How would your protoype differ?<br><br>Concluding question: Is there anything else you wish we would have asked you about?

Researcher Guide

Thank you for agreeing to take part in this focus group discussion. We would like to add some ground rules before going further. We recognize you are from diverse backgrounds and work in varied settings. We are eager to hear from all of you and all opinions and ideas and perspectives are of value to us. Can we agree each person will raise their hand to speak? You can also use the chat window for additional comments or clarifying questions, if necessary. We hope to have you share your thoughts and respond to the ideas and perspectives of others in a way that is respectful, honest, and comfortable. We would like to record this meeting, is that okay with you?

Start with overview of the project:
We are here today to get your perceptions about AI-enabled point of care obstetric ultrasound (POCUS) in LMICs and dive a little deeper into research priorities. This is an emerging field whereby AI is being used to analyze images captured by blind sweeps of the pregnant woman's abdomen. The output could be identification of conditions that might warrant further assessment, attention or higher level of care, such as fetal position, multiple gestation, placental abnormalities, etc.

Before we start, do you have any questions?
Let's go around and introduce ourselves:

- *First name only*
- *How many years in field*
- *Type of facility they currently work at and approximately how many antenatal clients they see a month (if provider-focused group or interview)*

**note that identifying information (e.g., your name) will not be linked to any data presented.*

**Domain**	**Prompts/questions**
Current practice for ultrasound	Let's start by talking about current research activities related to use of ultrasound. (POCUS)<br>• Where and how does obstetric US get performed? (Do you have POCUS or do you send clients to another unit?)<br>○ Probe: referral challenges<br>• ANC vs. maternity unit<br>• Perceptions on how it impacts your workflow, workload, etc.<br>• Perceptions on how it impacts your provider-patient dynamic
Existing knowledge and experience with POCUS	For those of you who have POCUS in your setting<br>• What has been the hardest part of using POCUS in your setting?<br>• Tell us about your training? What worked and what didn't work?<br>• What cadre of provider uses POCUS?<br>• Was certification required?<br>• Any mechanisms for quality control?<br><br>What are some issues related to POCUS adoption?<br>• Staff turnover<br>• Costs, supplies, machine maintenance etc.<br>• Women's demand<br>○ Probe: demand by trimester<br><br>How does using POCUS help you make decisions for clinical management of your patients? // Can you think back to the last time you performed an abnormal scan and tell me about what and you patient did to navigate it?

**Prototype Introduction:** Device Description prior to AI-related questions	Disclaimers:<br>1. This AI-enabled component is not currently available in your country<br>2. We are not here to advocate for the technology, but we are here to understand your opinions about its potential implementation<br>3. This technology is not yet cleared for use in any other country however the BMGF is putting effort into making it accessible. We are sharing our findings with them but do not represent BMGF and encourage you to be honest about your opinions about this technology.<br>We will now show you/ tell you about how the technology works (PPT/ video/ etc)<br><br>*The AI-enabled ultrasound probe is a portable handheld device with screen (smartphone or tablet) that will enable a provider to conduct blind sweeps (three vertical and three horizontal sweeps) across the belly. These sweeps are guided by real-time correction by the device (when the sweep is sufficient, the provider is guided to the next step. If the sweep is not sufficient, the provider is guided to redo the sweep correctly). The image is automatically made available and visible on the screen, along with key information that is automatically generated by AI algorithms. This information will include:* ***gestational age dating, singleton or multiples, placenta position, amniotic fluid, and fetal lie.***<br><br>• If asked about the cost: "Cost is certainly a consideration in the product development process, but it's too early in the product development process to share information on target pricing."
How might AI alter this experience?	Given the prototype that we just described, what are the potential benefits?<br>• How will the AI-component improve POCUS for you? For OB services for your patients?<br><br>You mentioned xyz problems with POCUS, how do you think AI will address or not address your earlier concerns with POCUS?<br>• Clinical: identification of complication, referral, case presentation<br>• Provider: turnover, skills retention, ease of use, behavior change<br>• Women: ANC utilization, fear<br><br>Given the prototype that we just described, what are some unintended consequences that might occur with the introduction of AI-enabled POCUS?<br><br>What do you expect will be barriers to implementing or adopting this into your setting?<br><br>Would you try this prototype now if you had access to it?<br>• In what situations would it be most useful?<br>• What resources would need to be in place already in order to implement?<br>• What would be the ideal training approach/modality?<br><br>Given the prototype that we just described, how do you think your clients would respond to information provided by an AI-enabled US compared to the current mode of POCUS?<br>• How important is the image functionality to you and the women you see?<br><br>**What research questions/data would you want to see before you would try it?**
Other critical factors to consider	What other additional considerations do you think need to be made before or research completed in parallel with implementing the prototype described?<br>• Supply chain, cost<br>• Medical liability<br>• Polices or guidelines<br><br>The prototype that we described is what is currently under development. If you could choose, would you work on an AI device with different functionalities? How would your protoype differ?<br><br>Concluding question: Is there anything else you wish we would have asked you about?

Decision-Maker Guide

Thank you for taking part in this conversation. We would like to record this meeting, is that okay with you? Your name won't be linked to any answers you provide.

Start with introductions and overview of the project.

Domain	Prompts/questions
Introductory questions	1. Can you tell us a little about your role at [*name of institution/organization*]?<br>a. How long have you been there?<br>b. How does your work relate specifically to POCUS or AI?<br>2. Prior to us reaching out to you, had you heard about some of the emerging work related to AI and POCUS for obstetrics?<br>*a.* If no – provide a brief overview<br>b. If yes,<br>*i.* What devices are you familiar with? (*ask about any others under development)*<br>ii. What studies do you know about? *(ask about study design, outcomes, who is funding or implementing this work*)<br>iii. What applications are you aware of?
Perceptions about emerging field of POCUS/AI	3. Based on what you have heard or know about this field:<br>a. What are your perceptions of AI-enabled devices?<br>b. What do you perceive as advantages?<br>c. What are your concerns? Unintended consequences?<br>d. What other additional considerations do you think need to be made?<br>i. Training: certification, QA, training modality<br>ii. Tasking sharing/shifting, health system capacity, referral<br>iii. Policies/guidelines<br>iv. Cost, supplies, sustainability<br>v. Use of advanced technologies, cultural acceptability<br>vi. Unintended consequences, misuse/overuse<br>4. Given your background in [*device development, as a funder, policymaker or researcher*], what are your impressions about how far along the evidence is? *(pre-clinical, efficacy, effectiveness, implementation). W*hat would you like to see next in terms of research? (*prioritize questions that are relevant to person's background*)<br>a. Potential probes: efficacy/accuracy<br>i. Clinical outcomes to measure<br>ii. Process outcomes to measure (ANC utilization, referral)<br>iii. Trial designs or ideal settings<br>b. Potential probes: effectiveness<br>i. Pre-requisites for LMIC contexts: equipment, infrastructure, medicines, blood products, etc.<br>ii. Level of health system<br>iii. Provider cadre<br>iv. Feasibility and acceptability from various lenses – women, provider and systems<br>c. Potential probes: implementation research<br>i. Training approaches<br>ii. Integration with guidelines/practice<br>iii. Costing<br>iv. Package approach
**Prototype Introduction:** Device Description prior to AI-related questions	Disclaimers:<br>1. This AI-enabled component is not currently available in your country<br>2. We are not here to advocate for the technology, but we are here to understand your opinions about its potential implementation

<table>
<tr><td></td><td>3. This technology is not yet cleared for use in any other country however the BMGF is putting effort into making it accessible. We are sharing our findings with them but do not represent BMGF and encourage you to be honest about your opinions about this technology.
We will now show you/ tell you about how the technology works (PPT/ video/ etc)

The AI-enabled ultrasound probe is a portable handheld device with screen (smartphone or tablet) that will enable a provider to conduct blind sweeps (three vertical and three horizontal sweeps) across the belly. These sweeps are guided by real-time correction by the device (when the sweep is sufficient, the provider is guided to the next step. If the sweep is not sufficient, the provider is guided to redo the sweep correctly). The image is automatically made available and visible on the screen, along with key information that is automatically generated by AI algorithms. This information will include: gestational age dating, singleton or multiples, placenta position, amniotic fluid, and fetal lie.

Quick replies
1. If asked about the cost: “Cost is certainly a consideration in the product development process, but it’s too early in the product development process to share information on target pricing.”</td></tr>
<tr><td>AI-enabled challenges, benefits, remaining issues</td><td>What benefits do you envision with the prototype just described?
- Systems
- Providers
- Access

What resources would need to be in place already in order to implement?

How will the AI-component improve POCUS for you?
In what situations would it be most useful?

How do you think this prototype will change (or not) patient-provider interactions? Clinical decision making?
• Given the prototype that we just described, how do you think providers and women would respond to information provided by an AI-enabled US compared to the current mode of POCUS?

You mentioned xyz problems with POCUS, how do you think AI will address or not address your earlier concerns with POCUS?
• Clinical: identification of complication, referral, case presentation
• Provider: turnover, skills retention, ease of use, behavior change
• Women: ANC utilization, fear

Given the prototype that we just described, what are some unintended consequences that might occur with the introduction of AI-enabled POCUS?

What research questions/data would you want to see before its implementation?

Additional questions if applicable and if time:
• What would be the ideal training approach/modality?</td></tr>
<tr><td>Concluding</td><td>Is there anything else you wish we would have asked you about with regards to POCUS and/or AI-enabled POCUS?</td></tr>
</table>

This was great - thank you for taking part in this conversation. Our next steps are to summarize this and other group discussions so that we may create a roadmap of AI-enabled POCUS research and implementation. If you have any further questions or thoughts you would like to convey, feel free to reach out to us via email.
